# Good Fences: The Importance of Setting Boundaries for Peaceful Coexistence

**DOI:** 10.1371/journal.pone.0095660

**Published:** 2014-05-21

**Authors:** Alex Rutherford, Dion Harmon, Justin Werfel, Alexander S. Gard-Murray, Shlomiya Bar-Yam, Andreas Gros, Ramon Xulvi-Brunet, Yaneer Bar-Yam

**Affiliations:** New England Complex Systems Institute, Cambridge, Massachusetts, United States of America; Universidad de Zarazoga, Spain

## Abstract

We consider the conditions of peace and violence among ethnic groups, testing a theory designed to predict the locations of violence and interventions that can promote peace. Characterizing the model's success in predicting peace requires examples where peace prevails despite diversity. Switzerland is recognized as a country of peace, stability and prosperity. This is surprising because of its linguistic and religious diversity that in other parts of the world lead to conflict and violence. Here we analyze how peaceful stability is maintained. Our analysis shows that peace does not depend on integrated coexistence, but rather on well defined topographical and political boundaries separating groups, allowing for partial autonomy within a single country. In Switzerland, mountains and lakes are an important part of the boundaries between sharply defined linguistic areas. Political canton and circle (sub-canton) boundaries often separate religious groups. Where such boundaries do not appear to be sufficient, we find that specific aspects of the population distribution guarantee either sufficient separation or sufficient mixing to inhibit intergroup violence according to the quantitative theory of conflict. In exactly one region, a porous mountain range does not adequately separate linguistic groups and that region has experienced significant violent conflict, leading to the recent creation of the canton of Jura. Our analysis supports the hypothesis that violence between groups can be inhibited by physical and political boundaries. A similar analysis of the area of the former Yugoslavia shows that during widespread ethnic violence existing political boundaries did not coincide with the boundaries of distinct groups, but peace prevailed in specific areas where they did coincide. The success of peace in Switzerland may serve as a model to resolve conflict in other ethnically diverse countries and regions of the world.

## Introduction

Efforts to resolve conflicts and achieve sustained peace are guided by perspectives about how conflict and peace are based in interpersonal and intergroup relationships, as well as historical, social, economic and political contexts. Recently, we introduced a complex systems theory of ethnic conflict that describes conflicts in areas of the former Yugoslavia and India with high accuracy [1]. In this theory, details of history, and social and economic conditions are not the primary determinants of peace or conflict. Instead the geographic arrangement of populations is key. Significantly, our theory points to two distinct conditions that are conducive to peace – well mixed and well separated. The first corresponds to the most commonly striven for peaceful framework: a well integrated society [2]. The second corresponds to spatial separation, partition and self determination – a historically used but often reviled approach [3]. Here we consider a more subtle third approach, that of within-state boundaries in which intergroup cooperation and autonomy are both present. The success of this approach is of particular importance as the world becomes more connected through international cooperation. As illustrated by the European Union, the role of borders as boundaries is changing.

**Figure 1:**
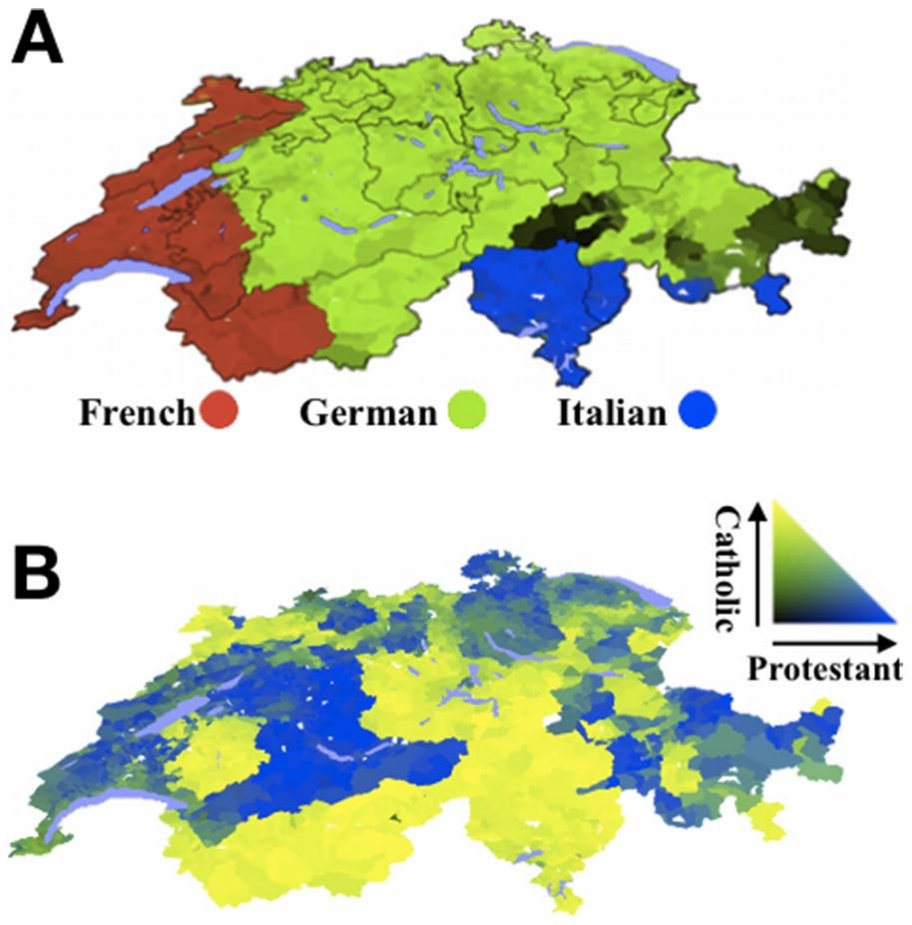
Maps of Switzerland showing the 2000 census proportion of (A) linguistic groups, (B) Catholics and Protestants (Mercator projection).

In order to evaluate the role of within-state boundaries in peace, we considered the coexistence of groups in Switzerland. Switzerland is known as a country of great stability, without major internal conflict despite multiple languages and religions [4], [5]. Switzerland is not a well-mixed society, it is heterogeneous geographically in both language and religion (Fig. 1). The alpine topography and the federal system of strong cantons have been noted as relevant to coexistence; their importance can be seen in Napoleon's statement, after the failure of his centralized Helvetic Republic, that “nature” had made Switzerland a federation [6]–[8]. But the existence of both alpine and non-alpine boundaries between groups and the presence of multiple languages and religions within individual cantons suggest partition is not essential for peaceful coexistence in Switzerland. In identifying the causes of peace, the literature has focused on socio-economic and political conditions. These include: a long tradition of mediation and accommodation; social cleavages that “cross-cut” the population rather than coincide with each other; unwritten and written rights of proportionality (fairness) and cultural protectionism; a federal system with strong sub-national units; a civil society that fosters unity; direct democracy through frequent referenda; small size; historical time difference between cleavages in language and religion; neutrality in international warfare; and economic prosperity [4]–[6], [9]–[13]. Geography plays an unclear, presumably supporting, role in these frameworks. The analysis of coexistence in Switzerland is also part of a broader debate about whether social and geographical aspects of federalism promote peace or conflict [15].

In this paper we analyze the geographical distribution of groups in Switzerland based solely upon the hypothesis that spatial patterns formed by ethnic groups are predictive of unrest and violence among the groups [1]. The model also allows that topographic or political boundaries may serve as separations to promote peace [1], [16]. We test the ability of the theory to predict peaceful coexistence in the context of internal country boundaries in Switzerland. Where explicit boundaries do not exist, such as in mixed cantons where alpine boundaries are absent, violence might be expected, and the results of the model in these areas serve as a particularly stringent test of the theory. In most such cases violence is not predicted, consistent with what is found. In one area a significant level of violence is predicted, and in fact violence is actually observed. The analysis sheds light on the example of Switzerland as a model for peaceful coexistence. The former Yugoslavia serves as a contrasting example of widespread violence. The theory also correctly identifies areas of conflict and areas of peace in the former Yugoslavia. The precision of the results provides some assurance of the usefulness of the theory in planning interventions that might promote peace in many areas of the world.

## Geographical Distribution Theory

The geographical distribution theory [1] is independent of the specification of the individual types – consistent with a universality of type behavior. Violence arises due to the structure of boundaries between groups rather than as a result of inherent conflicts between the groups themselves. Even though diverse social and economic factors trigger violence, it occurs when the spatial population structure creates a propensity to conflict, so that spatial heterogeneity itself is predictive of local violence. The local ethnic patch size serves as an “order parameter”, a measure of the degree of order of collective action, to which other aspects of behavior are coupled. The importance of collective behavior implies that ethnic violence can be studied in the universal context of collective dynamics, where models can identify how individual and collective behavior are related.

As we consider it here the analysis is applicable to communal violence and not to criminal activity or international warfare. In highly mixed regions, groups are not large enough to develop strong collective identities, or to identify public spaces as associated with one or another cultural group. They don't impose upon and are not perceived as a threat to the cultural values or social/political self-determination of other groups. At the other extreme, when groups are larger than the critical size, they typically form self-sufficient entities that enjoy local sovereignty. Partial separation with poorly defined boundaries fosters conflict. Violence arises when groups are of a geographical size that they are able to impose cultural norms on public spaces, but where there are still intermittent violations of these rules due to the overlap of cultural domains. Hence, we expect violence to arise when groups of a certain characteristic size exist. The model depends on population geography and not on the mechanisms by which the population structure arose, which may include individual choice as well as internally or externally directed migrations. The use of population geography, determined by census, to predict violence may work well because geography is an important aspect of the dimensions of social space, and other aspects of social behavior (e.g., isolationism, conformity, as well as violence) are correlated to it.

Physical boundaries such as mountain ranges and lakes or national and subnational political boundaries that establish local autonomy may prevent the violations of cultural norms that cause friction between groups and promote self-determination, inhibiting the triggers of violence. By creating autonomous domains of activity and authority, the boundaries shield groups of the characteristic size from each other when they correspond with their geographical domains.

Mathematically (see Methods), evaluation of the model begins by mapping census data onto a spatial grid. In this work we included the fraction of every population type on each site. The expected violence is determined by detecting patches consisting of islands or peninsulas of one type surrounded by populations of other types. These features are detected by pattern recognition using the correlation of the population for each population type with a template (filter) that has a positive center and a negative surround. The template used is based on a wavelet filter [1], [17], [18]. The wavelet filter is a conventional filter for identifying the geographic size of spatial regions. It corresponds also to the on-center off-surround detection elements of spatial size of regions in the human retina. Other methods that identify the size of groups provide the same results. We also find that the results are insensitive to the specific values of the parameters and therefore the analysis is highly robust to variations in the methodology and its parameters. The diameter of the positive region of the wavelet, i.e., the size of the local population patches that are likely to experience violence, is the only essential parameter of the model. The parameter is to be determined by agreement of the model with reports of violence, and results were robust to varying the parameter across a wide range of values. To model the effect of boundaries, we include only the populations within each of the autonomous areas to determine the expected violence. Where boundaries are incomplete, as might be the case for mountains, lakes and convoluted political boundaries, we include only the populations that are in line of sight through gaps or past ends of boundaries to determine the expected violence within a region. For each location, populations past boundaries of the line of sight are replaced by neutral populations. The result of the correlation of population with the wavelet filter is a single value at each location, the theoretical “propensity to violence”, and the locations of expected violence are obtained by applying a threshold to that value. The location of groups of a certain size is indicative of a violence-prone area, but the precise location of violence is not determined. We construct maps of the proximity of every location to the identified violence-prone groups, and the proximity of each location to reported violence. The correlation of these maps tests the ability of the theory to predict violence prone and peaceful regions. The model was validated without boundaries [1] by applying it to the former Yugoslavia, yielding correlations of up to 0.89. The results were robust to varying the characteristic length between 18–60 km, and thresholds in the range 0.2–0.4. Our revised method with fractional population values on every site gave similar results with correlations of up to 0.87.

## Results

### Switzerland

We now consider the linguistic and religious groups in Switzerland, each in turn. Initial analyses and the historical sequence of boundary formation suggested considering topographical barriers when discussing language groups, and political barriers when considering religious groups. The geography of languages primarily reflects the extent of invasions prior to the existence of current political boundaries and has remained stable in most areas for over a thousand years [5]. The modern state was established afterwards, and religious conflict played a role in establishing the internal political boundaries [5]–[7]. Census data were obtained for 2634 municipalities (communes) in Switzerland (bfs.admin.ch), yielding a high spatial resolution.

#### Language and topographical barriers

**Figure 2:**
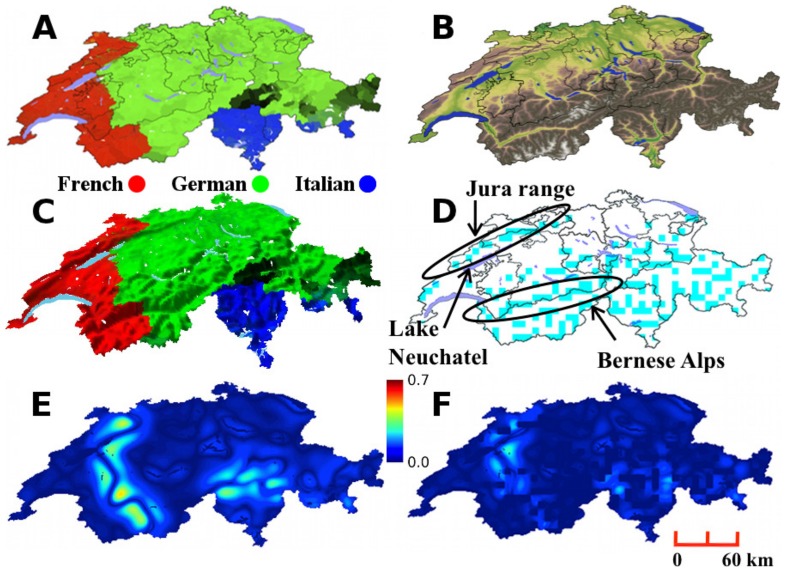
Linguistic groups and topographical boundaries in Switzerland. Maps of Switzerland showing (**A**) proportion of linguistic groups according to the 2000 census, (**B**) elevation within Switzerland, (**C**) overlay of linguistic groups onto a digital elevation model, and (**D**) topographical features including lakes (blue) and ridges extracted using edge detection (cyan). Comparison of calculated propensity (color bar) to violence between linguistic groups without (**E**) and with (**F**) the inclusion of topographical features as boundaries using a characteristic length scale of 24 km. Mercator projection, except C which is the Europe Albers projection. The distance scale is approximate.

**Figure 3:**
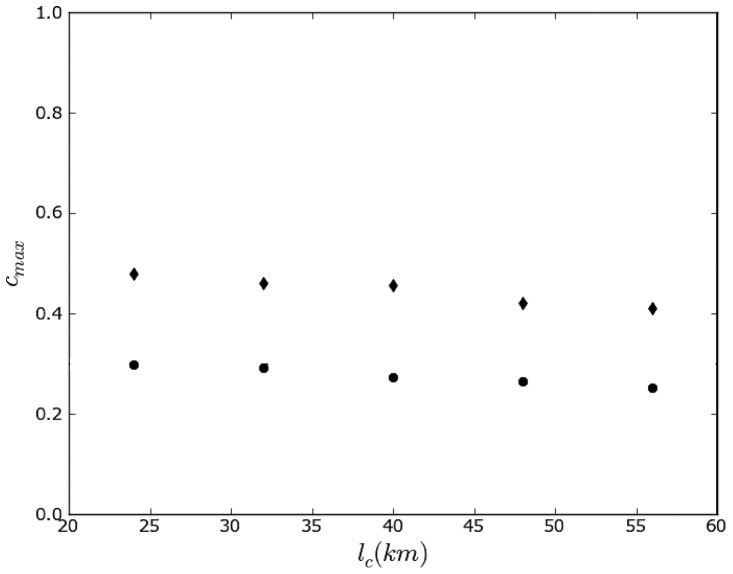
Effect of topographical boundaries on the propensity to linguistic conflict in Switzerland. Maximum level of the propensity to violence between linguistic groups in Switzerland as calculated in the model as a function of the characteristic length scale. The calculation is performed with effect of topographical boundaries (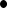) and without effect of topographical boundaries (⧫).

We study the three main language groups – German, French and Italian (Fig. 2A) – which together comprise 91% of the total population in the 2000 census (Romansh, the fourth official language, accounts for less than 2%). We considered only the effect of physical boundaries due to lakes and mountain ranges (Fig. 2, B and C). We determined the presence of topographical boundaries using an edge detection algorithm on topographical heights (Fig. 2D). This process identifies where there is a sharp change in height, i.e., a cliff, or steep incline, that runs for a significant distance forming a natural boundary. Elevation data with a spatial resolution of approximately 91 m [19] was coarsened to pixels of size 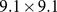 km. Edges were identified where there was an increase of more than 1.8 km in height over a distance of 9.1 km (11.5°) using a discretized Laplacian differential operator [20] with a mask size of a single pixel. The conclusions are robust to variations in the elevation angle (Methods). Calculations of the propensity to violence are shown in Fig. 2, E and F for the characteristic length of 24 km. Without boundaries, the correlation of the wavelet filter yields a maximum propensity to violence value of 0.48. With topographical boundaries the maximum propensity is reduced to 0.33. Results across filter lengths in the range 24–56 km, shown in Fig. 3 (maps in the methods section), consistently show that the propensity for violence is high for calculations without topographical boundaries and is dramatically reduced by their inclusion. Between the German and French-speaking areas the Jura mountain range and Lake Neuchatel serve as mitigating boundaries in the northwest, and the Bernese Alps are mitigating boundaries in the south. The interface between Lake Neuchatel and the Bernese Alps through the canton of Fribourg has no mitigating boundary, but is almost straight – neither side is surrounded by the other, so the propensity is not high. Between the Italian and German-speaking areas, the Lepontine Alps dramatically reduce the calculated propensity.

The Jura range is, however, a porous boundary, and the highest residual propensity is adjacent to it in the northwest of the canton of Bern, which, unique in Switzerland, is historically known to be an area of “intense” linguistically-based conflict, including arson, bombings and other terrorist tactics [13], [21]. We obtained a correlation higher than 0.95 between predicted and reported violence (Methods), consistent with the hypothesis of the model. Manifesting Swiss willingness to create political boundaries, the conflict led to a referendum, and in 1979 the modern-day canton of Jura was created out of part of the north of what was then the canton of Bern [7]. While the conflict underlying the unrest was linguistic, local votes led to separation by majority religion. However, conflict did not end, and a proposal to shift the French-speaking Protestant areas of Bern to join French-speaking Catholic Jura is currently being considered [22].

While the topographic boundaries are the historical boundaries between linguistic groups, and canton boundaries may not be able to play the same role, the creation of the canton of Jura suggests also considering the role of political boundaries in linguistic separation. Calculations including the canton boundaries reduce the maximum calculated propensity to violence to 0.22, just within the range of thresholds at which violence may be expected, which may be a context for intermittent violent outbursts [1]. The uncertainty about the role of canton boundaries for linguistic separation prevents drawing firmer conclusions.

Our results suggest that a calculated propensity to violence using a threshold in a range 0.2–0.4 (and consistent with a value of 0.3) should be considered for violence even under the social and political conditions prevailing in Switzerland. Remarkably, at these thresholds high correlations also are found in the former Yugoslavia. That is, similar values for the calculated propensity to violence correspond to actual violence in dramatically different social contexts.

#### Religious Groups and Political Barriers

**Figure 4:**
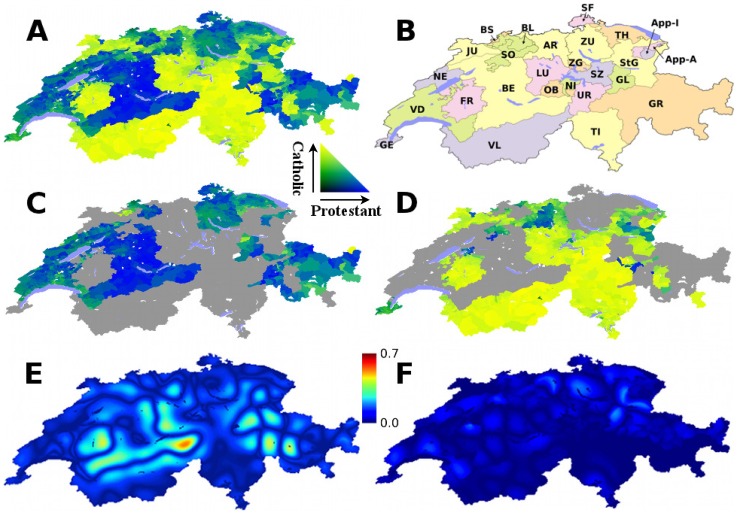
Religious groups and political boundaries in Switzerland. Maps of Switzerland (Mercator projection) showing (**A**) proportion of Catholic (yellow) and Protestant (blue) according to the 2000 census, (**B**) cantons, (**C**) and (**D**) cantons (and Graubünden circles) that are majority Protestant and Catholic respectively, using the same color map as A. Comparison of propensity to violence between religious groups without (**E**) and with (**F**) the inclusion of administrative boundaries using a characteristic length scale of 24 km. Propensity value scale is shown by color bar. Canton abbreviations are GE: Genève, SO: Solothurn, ZG: Zug, VL: Valais, BS: Basel-Stadt, GL: Glarus, VD: Vaud, BL: Basel-Landschaft, TI: Ticino, NE: Neuchatel, AR: Aargau, GR: Graubünden, FR: Fribourg, LU: Lucerne, App-A: Appenzell-Ausserhoden, BE: Bern, OB: Obwalden, App-I: Appenzell-Innerrhoden, JU: Jura, NI: Nidwalden, StG: St. Gallen, UR: Uri, SF: Schaffhausen, TH: Thurgau, SZ: Schwyz, ZU: Zurich.

The two main religious groups of Switzerland are Protestant and Catholic. The Swiss federal political system separates the country into 26 “cantons” and “half-cantons” considered as semi-autonomous political units (Fig. 4). Moreover, this schema is repeated within the largest canton by area, Graubünden, whose sub-cantonal divisions called circles (*kreise*) have a distinctive political autonomy [4], [12]. We obtained canton boundaries from mapping resources (www.gadm.org, www.toposhop.admin.ch). Circles boundaries were identified by district lists (www.gis.gr.ch). In the 2000 census, Roman Catholic and Protestant affiliations account for 77% of the total population. Less than 8% subscribe to other religions, and the remainder have no religious affiliation or did not specify one. Without boundaries, the maximum calculated propensity to violence is very high (0.57), and with political borders it is only 0.20. Without Graubünden circles, the propensity increases to a quite high 0.42, still well above the threshold.

**Figure 5:**
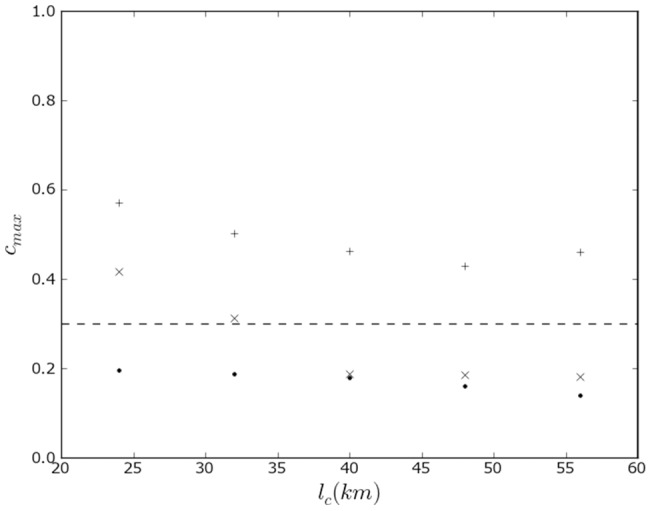
Effect of political boundaries on the propensity to religious conflict in Switzerland. Maximum level of the propensity to violence between religious groups in Switzerland as a function of characteristic length scale according to the model. Calculations are shown including the effect of canton boundaries and Graubünden circle boundaries (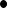), including the effect of canton boundaries only (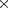), and without the effect of political boundaries (+). The dashed line represents the inferred threshold of propensity of violence in order for violence to occur.

Figure 5 plots the maximum propensity to violence as a function of length scale with canton and Graubünden circle boundaries, with canton boundaries only, and without political boundaries. The corresponding maps are shown in the methods section. The effect of canton boundaries is important across all length scales, that of the circles in Graubünden is important at the smaller length scales. This result specifically suggests that length scales of 24–32 km correspond to a geographical group size that is susceptible to violence. Because of a 10% decline in religious affiliation in recent years, we considered also the 1990 census, with similar conclusions (see Methods).

The separation of religions by canton is apparent geographically and historically. Autonomy within cantons and Graubünden circles has been established to prevent conflict. Consistent with the historical experience, the model results imply that without these boundaries violence would be expected, but with them it is not. In some cases the area of a canton includes small enclaves embedded in another canton whose majority religion corresponds to the canton to which they belong. Still, there are exceptions to the separation of religions by canton. In each case the geography is sufficient to limit the propensity to violence. For example, there is an area of Protestant majority in the far north of the Catholic canton of Fribourg. It is, however on a long appendage and therefore is not surrounded by Catholic areas, and so has a low propensity to violence according to the analysis. Historical evidence is found in conflict in the 1500s [7]. The Reformation led to cantons adopting a Protestant or retaining a Catholic identity. A brief war resulted in a peace treaty that established religious freedom by canton. The canton Appenzell was split by religious differences into two “half-cantons”, Innerrhoden and Ausserrhoden. The political independence of circles (*kreise*) in Graubünden also provided religious autonomy [12]. The intentional formation of political boundaries in regions that would have violence according to the model, and the subsequent model propensity to violence below the threshold associated with a lack of actual violence are consistent with the hypothesis on the role of boundaries in peaceful coexistence.

### Yugoslavia

**Figure 6:**
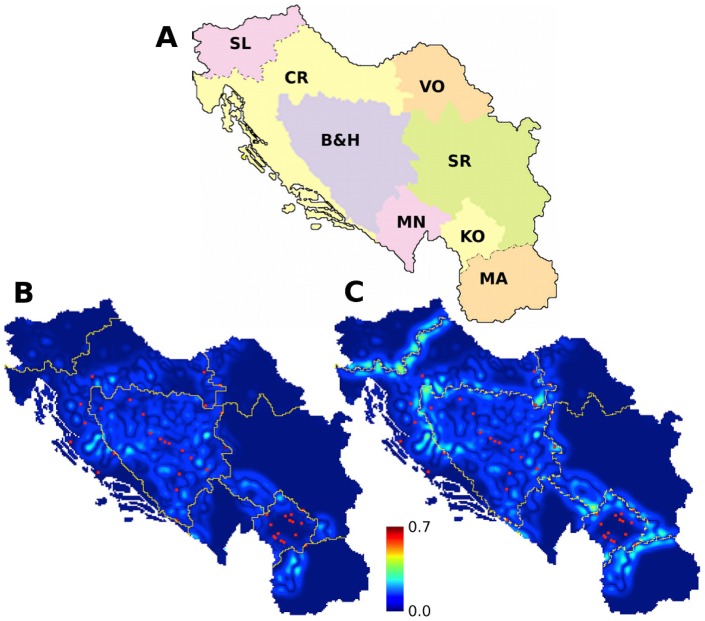
Ethnic groups, political borders, and topographical boundaries, in the former Yugoslavia. (**A**) Map of the area of the former Yugoslavia showing administrative provinces. Propensity to violence calculated without (**B**) and with (**C**) administrative boundaries, using a characteristic length of 21 km. Locations of boundaries are shown on both plots as solid and dashed yellow lines respectively. Sites of reported violence are shown as red dots [18]. Spurious violence is predicted along the borders of Slovenia and Macedonia when boundaries are not included. Province labels are: SL: Slovenia, CR: Croatia, VO: Vojvodina*, B&H: Bosnia & Herzegovina, SR: Serbia, MN: Montenegro, KO: Kosovo*, MA: Macedonia. (*Autonomous administrative provinces of Serbia.).

Our modified method including boundaries was tested on the previous case study of Yugoslavia, consisting of the combined area of Croatia, Bosnia, Serbia and Montenegro. Topographical boundaries reduce the maximum propensity from 0.63 to 0.57, and administrative borders to 0.56. The correlations of predicted and reported violence changes were insignificantly lower, with correlations of 0.86 and 0.85, respectively. That political boundaries do not have a greater impact on the calculated violence implies that they do not align with the geographical boundaries between groups. We also extended the area to include Macedonia and Slovenia, parts of the Socialist Federal Republic of Yugoslavia before gaining independence (Fig. 6). With the political boundaries the correlation is still 0.85; however, when political boundaries are not included, the correlation is reduced considerably to 0.72. The lower correlation is specifically due to a high calculated propensity to violence along the borders of Slovenia with Croatia, and of Macedonia with Serbia and Kosovo. These areas, however, were peaceful – consistent with the predictions when boundaries are included. Our results suggest that these political borders were instrumental in reducing ethnic violence, whereas the violence in other areas of Yugoslavia was not prevented because of poor alignment of borders with population groups. These results are robust to variations in both the size of the wavelet filter and the threshold of violence applied.

## Discussion

### Summary of model comparisons with the data

We briefly summarize seven categories of distinct sucessful comparisons between model predictions and the observed data that are contained in the results.

Our examination of linguistic and religious groups in Switzerland included cases where violence is predicted without the presence of boundaries, but is mitigated by the consideration of topographical and political boundaries appropriate to linguistic and religious groups, respectively.

1. Topographical boundaries reduced violence between linguistic groups. This occurred along (a) Alpine boundaries of the Swiss Alps between German-speaking and Italian-speaking populations, (b) Alpine boundaries between German-speaking and French-speaking populations, and (c) Jura range boundaries between German-speaking and French-speaking populations.
2. Political boundaries reduced violence between religious groups. This is the case both for (a) canton boundaries and for (b) circle boundaries in the canton of Graubünden.
  Our analysis also identified locations in which our model does not predict violence despite linguistic or religious heterogeneity and no explicit boundaries.
3. The straightness of the boundary prevents violence between linguistic groups in Fribourg/Freiburg.
4. Isolation of a Protestant population on an appendage from the Catholic majority prevents violence in Fribourg/Freiburg.
  We also identified one area at the highest level of calculated residual propensity to violence and it corresponds to an area of unresolved historical conflict.
5. The northeastern part of the canton of Bern is the location of both the highest prediction of propensity to violence, and a real-world history of intergroup tension. The unique condition of the conflict in this part of Switzerland and its correspondence to the prediction by the model provides additional confirmation of the model.
  Considering the predicted and reported violence in the former Yugoslavia also demonstrated the importance of the boundaries which coincide with ethnic divisions.
6. Political boundaries between Slovenia and Macedonia and the other countries of the former Yugoslavia prevent violence along their borders.
7. The borders between the countries of Croatia, Bosnia, Serbia and Montenegro were not aligned with the boundaries between ethnic groups and so were ineffective at reducing violence.

### Conclusions

This work is part of a broader effort to use new methods for quantitative analysis of patterns of violence and their prevention [23]–[31]. There is also interest in ethnic group interactions across national borders [32]–[34]. More generally, in recent years there have been increasing efforts to understand the causes and enabling conditions for civil war and ethnic conflict [36]–[120] (see also supplementary materials of Ref. [1]). These efforts include examinations of geography and other structures within countries [50]–[67] as well as the effects of transnational geography [68]–[79]. Extensive analysis explores the role of political structures, particularly federalism, in enabling or preventing civil and ethnic conflict [80]–[99]. Research has begun to include quantitative studies and modeling to understand human behavior and conflict [100]–[104]. A body of research examines Switzerland regarding the presence or absence of tensions and possible causes [105]–[120].

We have shown that groups that are not well-mixed but are geographically separated by natural or political boundaries into autonomous domains are peaceful in both Switzerland and the former Yugoslavia. Our work clarifies the ambiguities of mixed languages and religions in Swiss cantons by showing that in most cases the natural geography of the populations conspires to lead to a low level of violence, so that additional boundaries were not necessary; where they were needed, as in Graubünden, they were established. The highest calculated propensity to violence is between linguistic groups in the northern part of the canton of Bern, where historically unresolved real world tensions actually exist. Our analysis indicates that both administrative and natural barriers can play a significant role in mitigating conflict between religious and linguistic groups. Historical evidence suggests that for religious groups the boundaries in Switzerland were created to provide autonomy to a group with a shared identity and avoid conflict among multiple groups. Ongoing efforts to reduce tensions in Bern include introducing new political boundaries. The many political, social and economic factors that play roles in reducing violence [4]–[6], [9]–[14] build on a strong foundation of geographical borders. Our analysis suggests that when partition within a country is viewed as an acceptable form of conflict mitigation, such partition can give rise to highly stable coexistence and peace.

## Methods

### Identifying the propensity to violence using a wavelet filter

The potential for conflict is quantified in our model using a wavelet filter [1], [17], [18], [35]. In essence, the filter evaluates the extent of the presence of a type in a circular area with a specified radius and subtracts from this the presence of the same type in a surrounding area. This results in cancellation if the same type is located in the surrounding area. Other types are all treated with the opposite sign causing cancellation if there are mixed populations of the first type with the others. Thus, the largest values are obtained for an island of one type surrounded by other types. Large values are also obtained for a peninsula of one type into a sea of other types. To evaluate the likelihood of violence at a particular location, we apply the filter, centered at that location, for each of the types. The likelihood of violence in that region is the maximum over all types. Unlike the earlier method [1], we included all population types on each site of a grid rather than basing calculations on an agent model. Mathematically the expression for the filter applied at a location 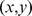, with the maximum taken over all types, is
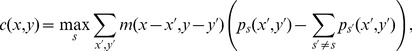
(1)which is a convolution of the fraction of the population of one type, 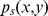 minus the fractional population of other types, with a wavelet,
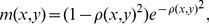
(2)where the scaled distance from the center is given by
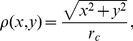
(3)the Euclidean distance divided by the radius of the wavelet, 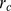, which is half of the diameter, 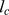, the model parameter identifying the size of groups that are likely to engage in conflict. The value of 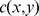 serves as a measure of the likelihood of violence in the vicinity of the location 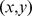. When performing statistical tests on the prediction of violence, we specify a threshold that distinguishes regions of violence from regions of non-violence according to whether 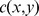 exceeds the specified threshold.

### Correlations of predicted and actual violence using proximity maps

The points of predicted violence and reported violence do not precisely overlap within the assumptions of the model. The model identifies regions of population that have high propensity to engage in violence not the specific location of the violence itself. Moreover, the news reports for conflict in Yugoslavia [1] identify towns in the vicinity of reported violence and not necessarily the actual site or region of violence. In some cases it is stated that violence extends throughout the region surrounding a city or town, or in a valley. Without detailed information of the boundaries of the region of violence, we mark only identified cities and towns. The reports may also be incomplete or biased. Thus, there are many reasons that the locations of model-predicted violence and reported violence should not correspond precisely. Moreover, the simplifying assumptions of the model ignore many factors influencing violence, such as variation in population density and geography, that affect local conditions. Despite these limitations, there is significant statistical agreement of the model with reported violence.

From a policy perspective, we assume that it is important to distinguish regions of likely violence from regions in which violence is not likely to take place. In order to compare predicted population areas and reported violence in quantitative statistical tests, we use the distance of each location from a region of violence as a measure of the effectiveness of the model. We test the model predictions against a null hypothesis reference model that predicts the same amount of violence (number of locations) as the reported violence but does so at random locations. By demonstrating a substantially better agreement, we can affirm that the locations of model-predicted violence are statistically correlated with the locations of reported violence.

We construct conflict maps of reported 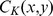, predicted 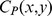, and randomized 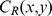 sites of violence. For the reported violence, cities or towns cited in news reports as particularly affected are included in 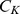 as well as their surrounding area within the map resolution limit. The propensity for violence predicted by the model 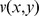 is associated to each point 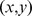 in the map using a wavelet filter. To identify which regions to report as having actual violence, we impose a cut-off value 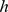 on the propensity values. All points with 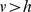, where 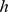 is a threshold parameter, are included in 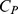. The results vary weakly over a range of values of 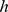. For the randomized case, we move the sites of reported violence within the territory of the state at random, generating multiple instances of randomized maps 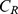.

In order to characterize agreement between model and reported distance to violence we construct conflict proximity maps 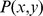, which are the minimum distance from any point 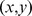 to a region of violence in the corresponding conflict map. Specifically, 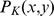 is based upon reported values and 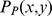 is based upon the predicted values. We then evaluate the agreement between the two conflict proximity maps by three statistical measures. The correlation (Pearsons correlation coefficient) is given by,
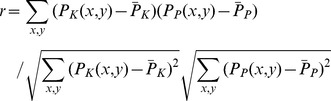
(4)where 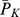 is the average of 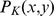, and similarly for 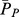. We also evaluate the normalized mean square error, which we subtract from one, and Spearman's non-parametric statistic [1], for comparison. We report results for the correlations, other statistical tests lead to similar conclusions.

### Topographical and administrative boundaries

We model both topographical and administrative boundaries within a country as preventing intergroup violence across them, similar to national boundaries in the earlier method [1]. A cliff separating a plateau from a plain is considered to be a barrier to movement between the upper and lower areas and thus serves as a boundary.

We generalize the previous method for incorporating boundaries to allow for partial boundaries and boundaries with gaps. Partial boundaries between areas within the country can arise due to mountains, lakes, or at convoluted political borders. For such boundaries, we consider the line of sight from a given location to identify the populations which impact on the propensity for violence at that location. Populations outside of the line of sight are not included as contributing to violence. Thus an effective map of populations as experienced at each site is constructed, determined by the specific orientation of any boundaries relative to that site. The areas which are blocked from sight are populated with a neutral population, the existing local proportions of the population. This better matches both the mixed and single type local populations than a single type. The local proportions were measured within a range of two characteristic lengths (wavelet diameters) of each site, considering only sites that are in a line of sight.

### Unpopulated sites

Some small areas are unpopulated. These and lake areas were treated as other sites, but the violence at these sites was set to zero. Only small differences arise if these unpopulated areas are treated differently.

**Figure 7:**
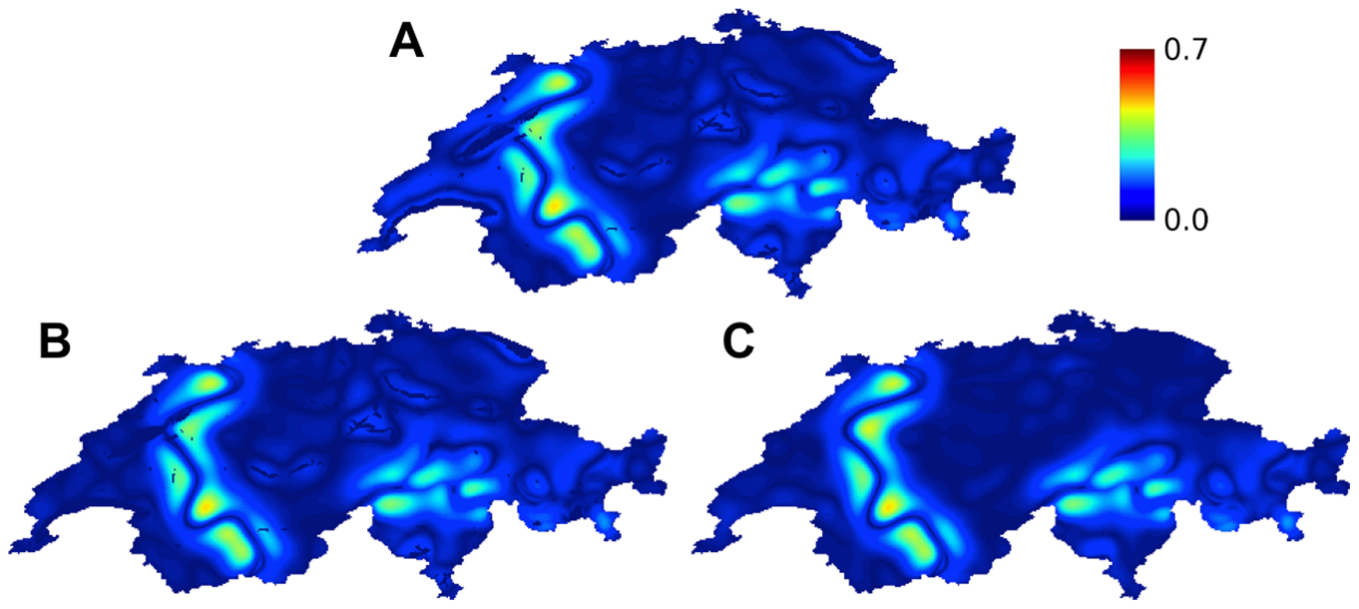
Sensitivity of the analysis for Switzerland to different treatment of unpopulated areas. Level of predicted violence between linguistic groups in Switzerland using a characteristic length scale of 24 km. Each panel represents results for a different treatment of lakes and unpopulated land areas: (**A**) including lakes and unpopulated land areas as empty sites; (**B**) including as barriers the lakes of Leman and Neuchatel; (**C**) interpolating a composition for all unpopulated sites from neighboring sites.

There are two types of unpopulated areas, land and water. Unpopulated land areas are treated as other land areas for the purpose of the calculations. After the calculation we set the propensity to violence in those locations to zero. The results were not affected significantly (Fig. 7). Water areas were treated similarly, with the exception that bodies of water that are large were considered to be topographical barriers, similar to mountains and cliffs. Specifically, we included the two largest lakes, Leman and Neuchatel, both of which have a length above 10 km, which is comparable to the range of characteristic length scales used to detect a propensity to violence.

### Census data for Switzerland

The commune composition used in our calculations was based on the census of 2000 and 1990 published by the Swiss Statistical Office. Where municipalities have merged, an aggregate of their previous constituent municipalities was taken. Three official languages we considered are French, German and Italian, which comprise 91% of the total population. The fourth official language, Romansch, is 2%. The religions considered are Roman Catholic and Protestant accounting for 77% of the total with less than 8% belonging to other religious groups and the remainder not subscribing to a religion or not specifying one. The 1990 census data is only readily available on a cantonal level. As described below we estimated the commune composition using the 2000 value and the change in the parent canton between 1990 and 2000.

### Elevation edges in Switzerland and Yugoslavia at different thresholds

**Figure 8:**
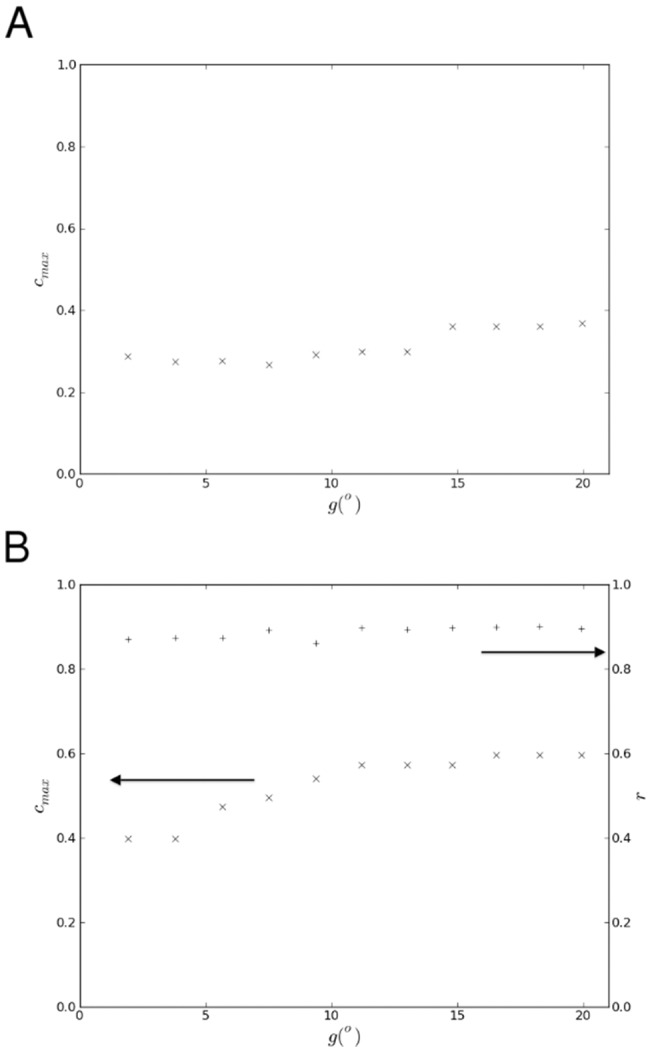
Sensitivity of the analysis to the topographical barrier threshold. The maximum propensity to violence between linguistic groups in as the threshold gradient for topographical barriers varies: (**A**) Switzerland; (**B**) Yugoslavia (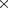, left axis). For Yugoslavia the correlation of predicted with reported violence is also shown (+, right axis).

We investigate the robustness of our analysis to variation of the gradient threshold that determines the presence of a topographical boundary and compare the results for linguistic groups in Switzerland. We also include here a similar comparison of the calculation of the impact of topographical edges on the conflict between ethnic groups within the former Yugoslavia. Figure 8A shows the variation of the maximum propensity to violence in Switzerland as the threshold gradient for geographical barriers varies. The propensity is robust to the variation across a range of angles. Still, as the gradient increases and barriers are removed the propensity to violence increases. The model results are consistent with the expectation that it is necessary to include geographical features as barriers in order to achieve agreement with the locations of actual reports of violence, and is consistent with the hypothesis that such barriers are effective in mitigating outbreaks of violence. Figure 8B shows the maximum propensity to violence calculated for the former Yugoslavia as a function of changes in the gradient threshold, and the resulting correlation of predicted and reported violence. The results show that while some variation in the maximum value of the predicted violence propensity occurs, it remains above the threshold for expected violence. The correlation with observed violence is not very sensitive to the gradient of the edges in elevation. This indicates that areas of predicted violence continue to be proximate to the areas of reported violence. Topographical features are not sufficiently steep or aligned with the boundaries of population groups to inhibit violence.

### Religion (1990 census) in Switzerland

In the main paper we reported the propensity for violence between religious groups for the 2000 census for the characteristic length of 24 km. During the 1990s there was a significant reduction in religious affiliation. We therefore considered also the 1990 census. The results are very similar to those of the 2000 census with maximum propensity without boundaries of 0.59 (compared to 0.57) reduced to 0.23 when including the political boundaries (compared to 0.20).

**Figure 9:**
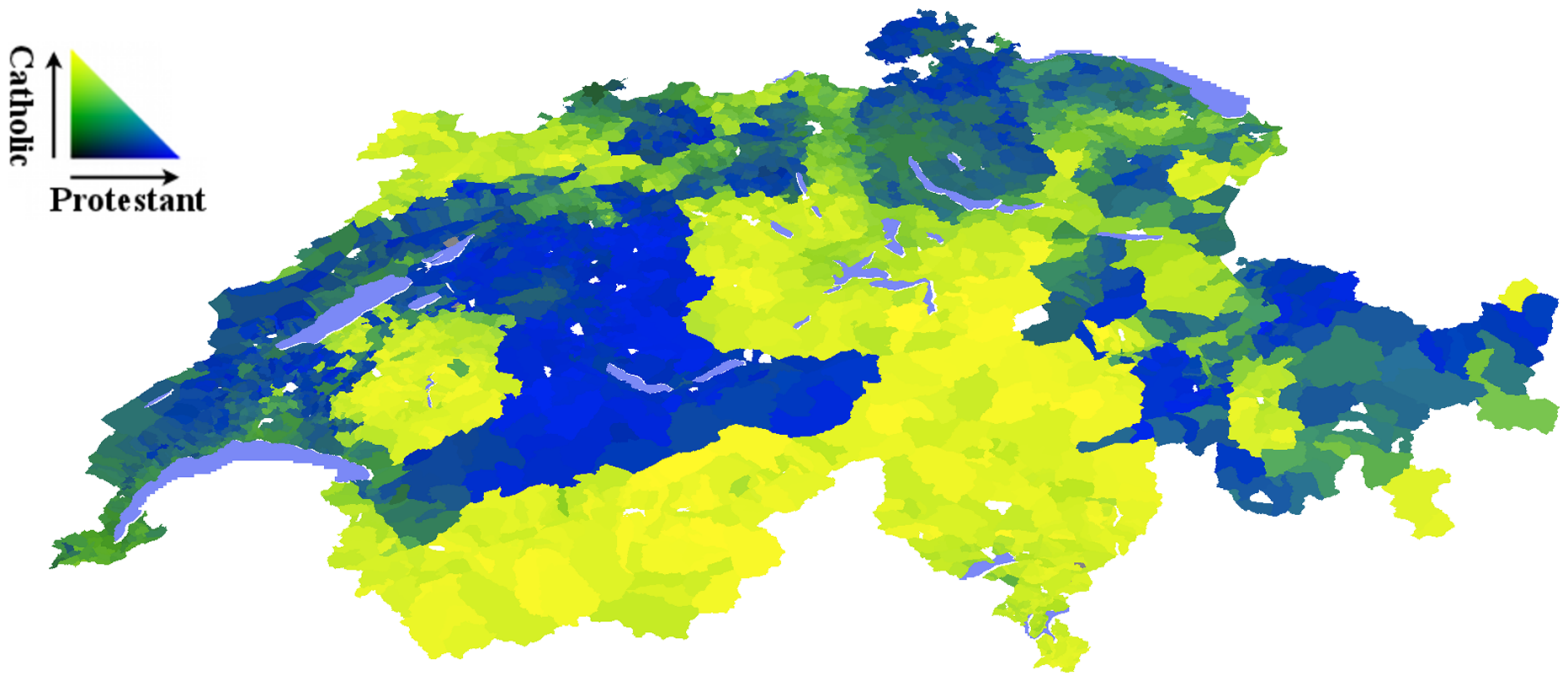
Religious groups in Switzerland according to interpolated 1990 census. Communes are colored according to proportion of Protestant (blue) and Catholic (yellow) as shown by color triangle.

**Figure 10:**
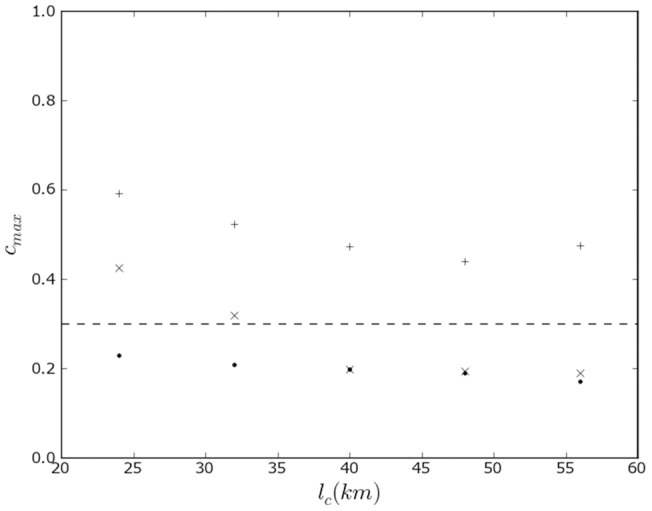
Effect of political boundaries on the propensity to religious conflict in Switzerland (1990 census). Maximum level of the propensity to violence between religious groups in Switzerland as a function of characteristic length scale according to the model. Calculations are shown including the effect of canton boundaries and Graubünden circle boundaries (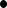), including the effect of canton boundaries only (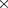), and without the effect of political boundaries (+). The dashed line represents the inferred threshold of propensity of violence in order for violence to occur. (Compare Fig. 5 for the 2000 census.).

In 1990 Roman Catholicism and Protestantism accounted for 87% of the population, 10% more than in 2000, and with only 9.5% identifying themselves as atheist or not specifying religious affiliation. The census for religions in Switzerland in 1990 is readily available only at a canton level resolution rather than the municipality level used in our calculations. We used the reduction of religious affiliation in the entire canton to estimate religious composition for each municipality in 1990. Explicitly:
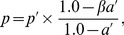
(5)where 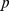 and 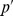 are the value of the municipal Catholic or Protestant proportion of the population estimated for 1990 and given for 2000, 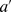 is the unaffiliated municipality population proportion in 2000, and
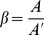
(6)is the ratio of unaffiliated canton population proportions, 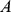 and 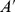, in 1990 and 2000. Fig. 9 is a map of the resulting religious affiliation. Fig. 10 shows the calculations of the propensity for violence for the 1990 census corresponding to the results for the 2000 census results shown in Fig. 5.

### Switzerland analysis at multiple length scales

In this section we show the results of calculations of the propensity for violence for languages and religions in Switzerland for a range of characteristic lengths, demonstrating the robustness of these results.

**Figure 11:**
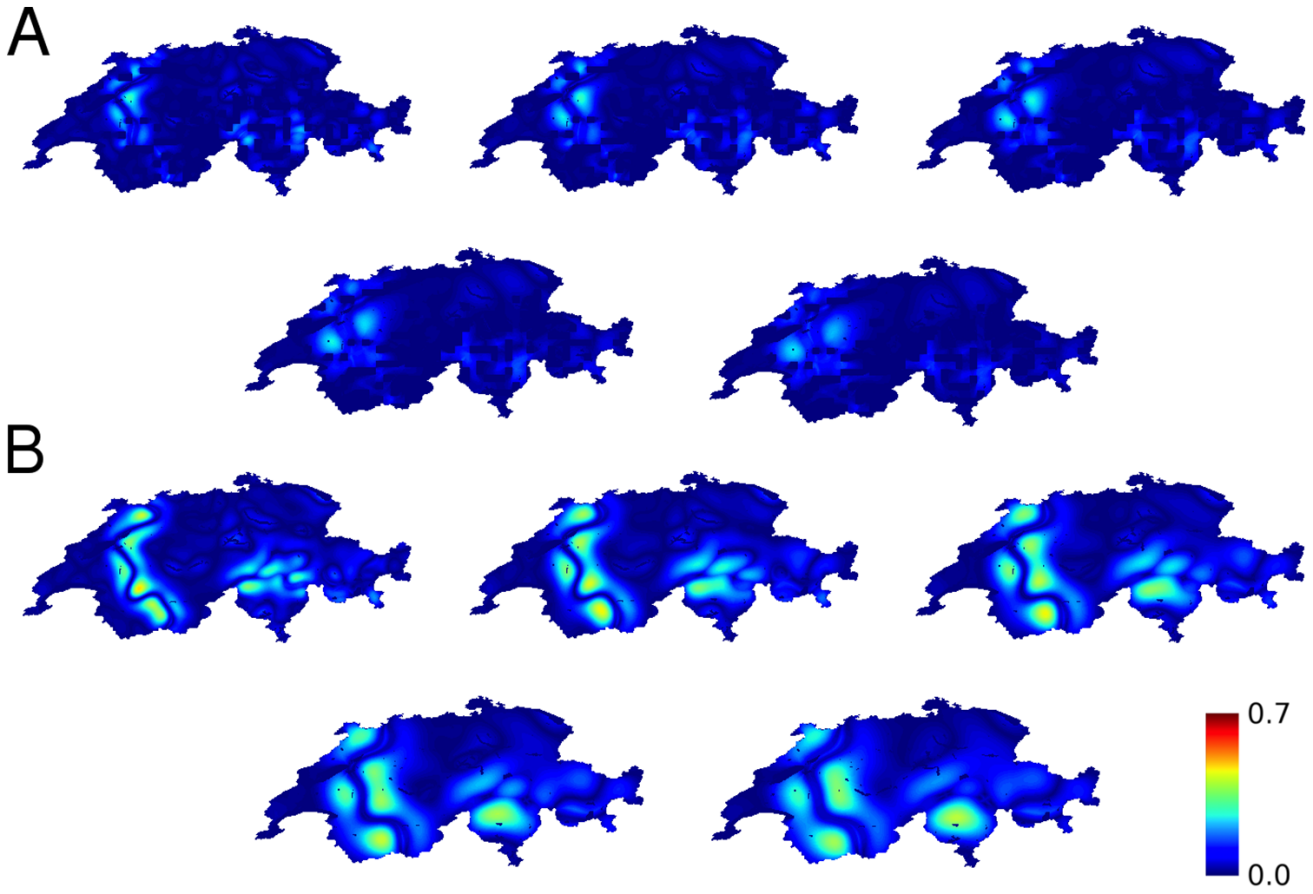
Calculated propensity to violence between linguistic groups in Switzerland. (**A**) including the effect of topographical boundaries, (**B**) without the effect of topographical boundaries. Characteristic lengths increase from left to right, first row then second row, with the values 24, 32, 40, 48, 56 km.

Calculations of the propensity to violence based upon linguistic groups are shown in Fig. 2, E and F for the characteristic length of 24 km. Results across characteristic lengths in the range 24–56 km, shown in Fig. 11, consistently show that the propensity for violence is high for calculations without topographical boundaries and is dramatically reduced by their inclusion.

**Figure 12:**
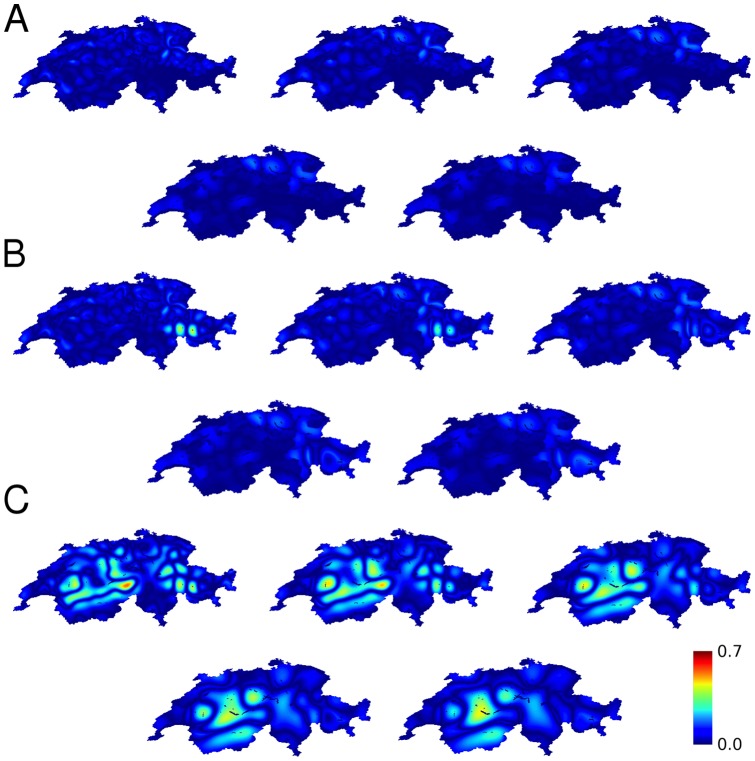
Calculated propensity to violence between religious groups in Switzerland (2000 census). (**A**) with political boundaries, including both cantons and Graubünden circles, (**B**) including only the effect of canton boundaries, (**C**) without the effects of political boundaries. Characteristic lengths increases from left to right, first row then second row, with the values 24, 32, 40, 48, 56 km.

**Figure 13:**
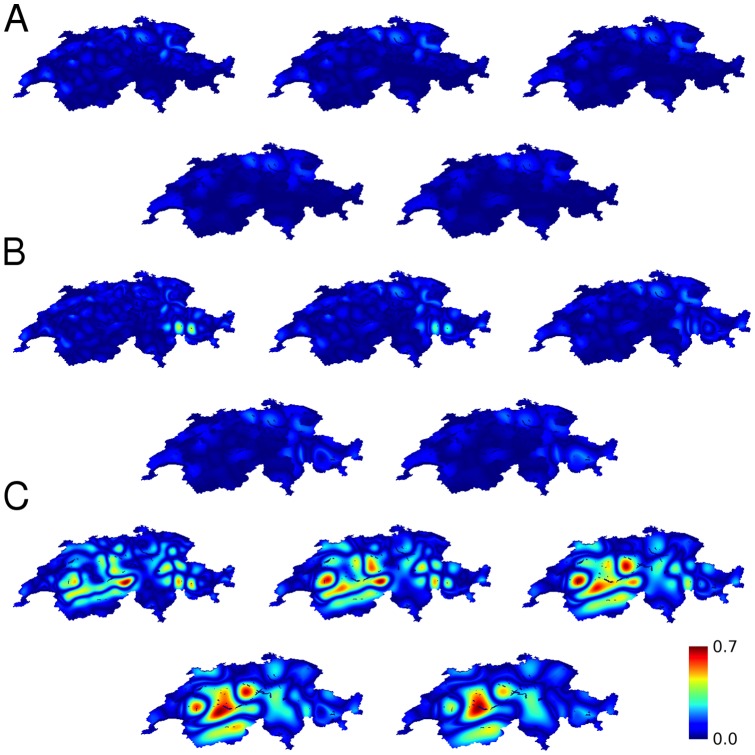
Calculated propensity to violence between religious groups in Switzerland (1990 census). As in Fig. 12 for the 1990 census. (**A**) with political boundaries, including both cantons and Graubünden circles, (**B**) including only the effect of canton boundaries, (**B**) without the effects of political boundaries. Characteristic lengths increases from left to right, first row then second row, with the values 24, 32, 40, 48, 56 km.

Calculations of the propensity to violence based upon religious groups are shown in the main text in Fig. 4 for the characteristic length of 24 km. Figure 5 plots the maximum propensity to violence as a function of length scale with canton and Graubünden circle boundaries, with canton boundaries only, and without political boundaries. The corresponding maps are shown in Fig. 12 (and for the 1990 census in Fig. 13). The effect of canton boundaries is important across all length scales, that of the circles in Graubünden is important at the smaller length scales. This result specifically suggests that length scales of 24–32 km correspond to a geographical group size that is susceptible to violence.

### Incidents of Bern/Jura violence in Switzerland

Unique in Switzerland in recent decades, the violence in the area of Bern/Jura based on linguistic conflict included targeted arson and bombings and a violent encounter between demonstrators. We performed an analysis of the correlation of reported violence with the location of highest propensity calculated by the theory, which is reduced by local geography compared to what would be expected without it. The resulting correlation is greater than 0.95. We note that the difficulty in relieving the conflict in the northern area of Bern is consistent with an expectation that political boundaries are used for inter-religious rather than inter-lingual conflict, for which purpose they may not be as well adapted.

Specific events, listed by location:

Glovelier – March 24, 1961, arson against a military arsenal. [121]; July 16, 1972, explosion of a military arsenal. [122] (http://www.bijube.ch/page-7207.html).

Les Auges – October 21, 1962, arson against a military barracks. [123] (http://www.bijube.ch/page-6210.html).

Bourrignon – March 26, 1963, arson against a military barracks. [123] (http://www.bijube.ch/page-6303.html).

Genevez – April 28, 1963, arson against a farm. [123] (http://www.bijube.ch/page-6304.html).

Montfaucon – July 18, 1963, arson against a farm. [123] (http://www.bijube.ch/page-6307.html).

Mont–Soleil – October 5, 1963, a house bombing against a leader of an anti-separatist group. [123], [124] (http://www.bijube.ch/page-6310.html).

Malleray – December 23, 1963, a bombing of a property of an anti-separatist group leader. [123]; October 20, 1987, arson against a shooting range. [125] (http://www.bijube.ch/page-6312.html, http://www.bijube.ch/page-8509.html).

Studen – February 27, 1964, bombing of a railway line. [123], [126] (http://www.bijube.ch/page-6402.html).

Delemont – March 12, 1964, bombing of a branch of the Cantonal Bank of Berne. [123]; March 4, 1966, government administration building attacked. [121] (http://www.bijube.ch/page-6403.html).

Saignelégier – November 20, 1965, arson against a hotel. [121]; On October 1, 1987, explosion of a munitions depot. [127] (http://www.bijube.ch/page-6511.html, http://www.bijube.ch/page-8710.html).

Mont-Crosin – May 29, 1966, arson against a hotel. [121] (http://www.bijube.ch/page-6605.html).

Cortébert – March 16, 1980, violent fighting between separatists and anti-separatists with stones, firecrackers, and flare guns. Demonstrators on both sides were injured. [128] (http://www.bijube.ch/page-8003.html).

Moutier – September 4, 1985 bombing of the district court. [129] (http://www.bijube.ch/page-8509.html).

Reussilles – September 11 and 23, 1993, arson against a munitions depot. [125], [127].

Perrefitte – October 21, 1987, bombing of a shooting range. [125] (http://www.bijube.ch/page-8710.html).

Büren – April 5, 1989, arson against a historic wooden bridge. [130] (http://www.bijube.ch/page-8509.html).

Montbautier – May 24, 1992, arson against a German-language school, previously vandalized. [131].

Courtelary – January 7, 1993, bombing of a house of an anti-separatist. [132], [133] (http://www.bijube.ch/page-9301.html.

Berne – January 7, 1993, premature explosion of a bomb in a car killing one person. [133] (http://www.bijube.ch/page-9301.html).

### Yugoslavia technical details

**Figure 14:**
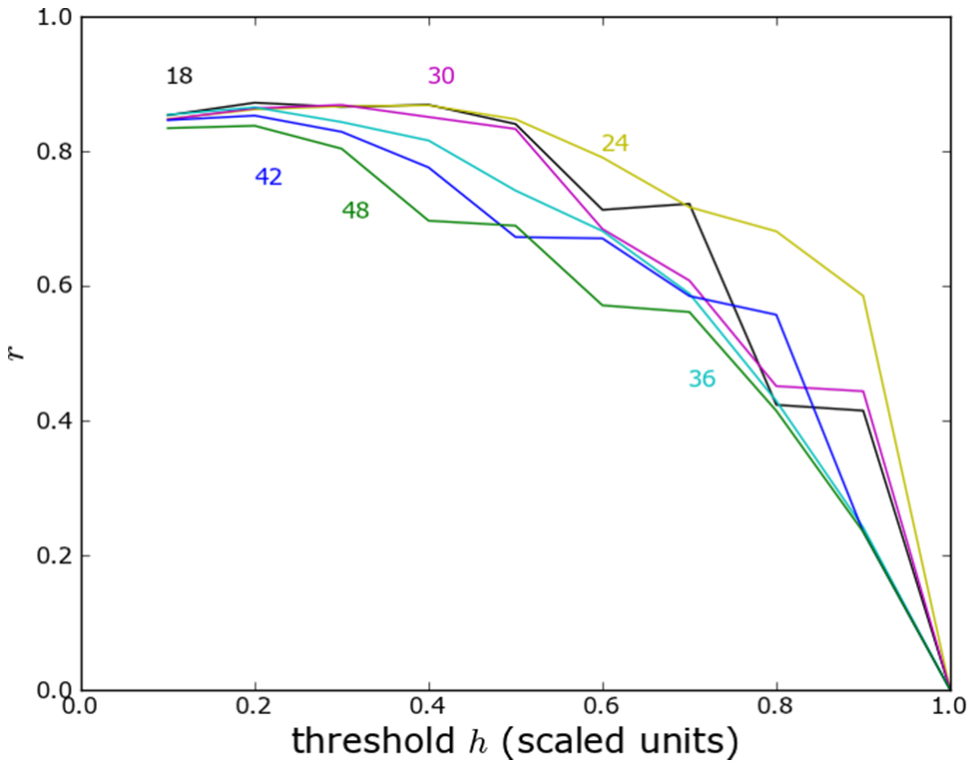
Correlation of proximity maps of predicted and reported violence in Yugoslavia without topographical or political boundaries. Results are shown as a function of threshold for violence divided by the maximum propensity for violence. Each curve is labelled by the characteristic length (km). (Compare with Figure S4.3 in Ref. [14].).

**Figure 15:**
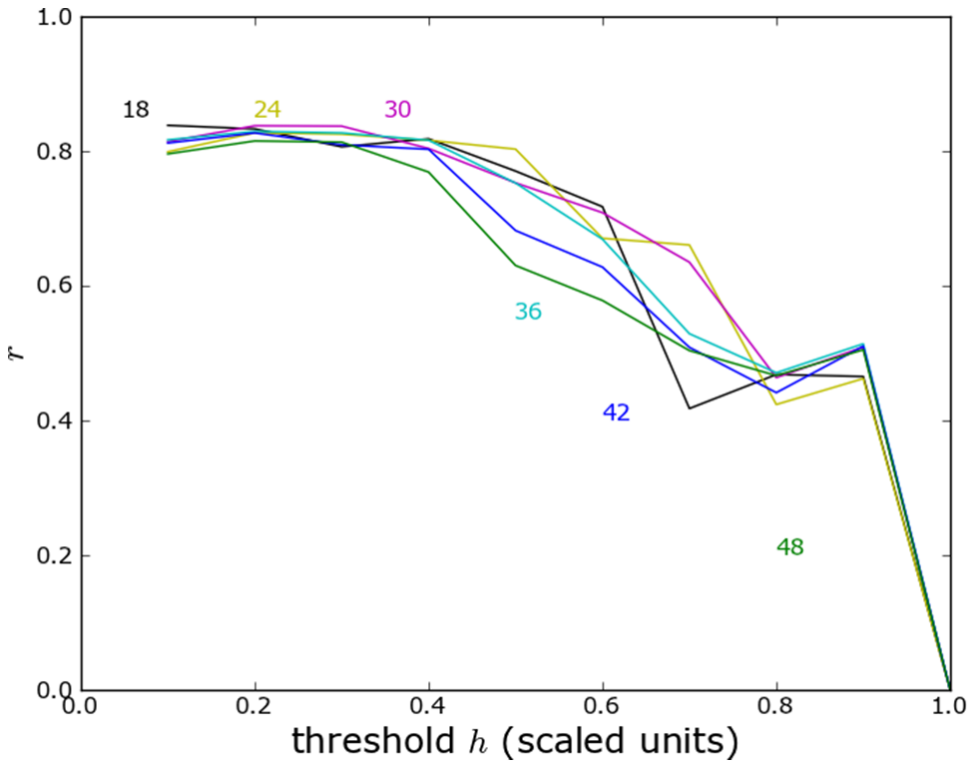
Yugoslavia correlation analysis with administrative boundaries. As in Fig. 14 but including the effects of administrative boundaries.

**Figure 16:**
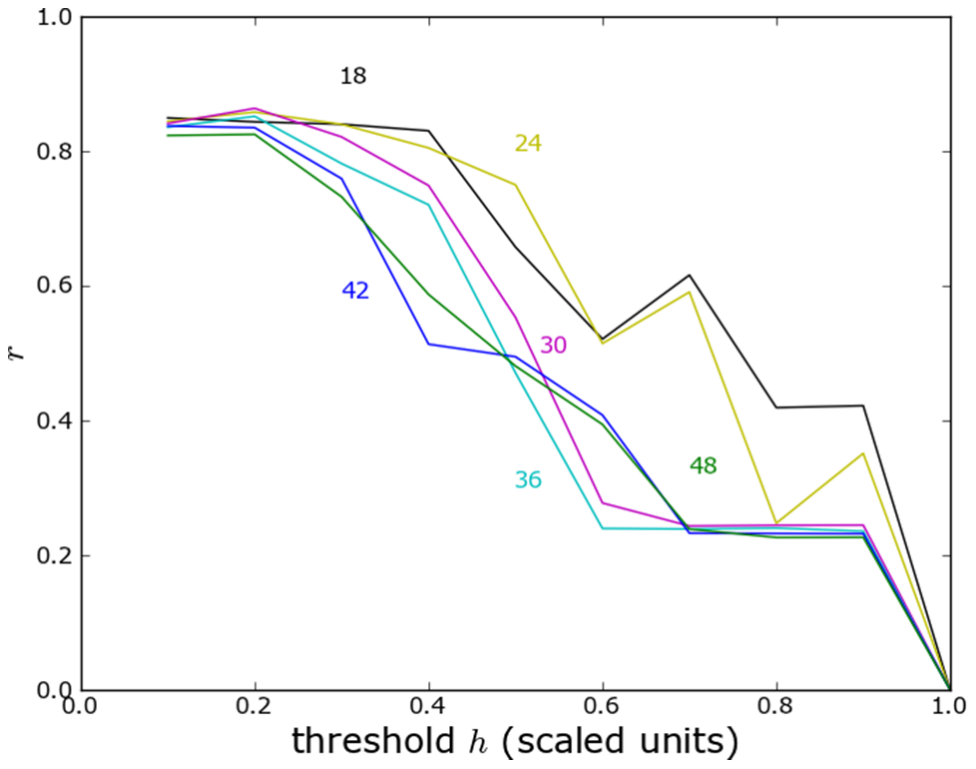
Yugoslavia correlation analysis with topographical boundaries. As in Fig. 14 but including topographical boundaries.

Here we show the correlation of predicted and reported violence for the former Yugoslavia without administrative or topographical boundaries (Fig. 14) with administrative boundaries (Fig. 15) and with topographical boundaries (Fig. 16).

**Figure 17:**
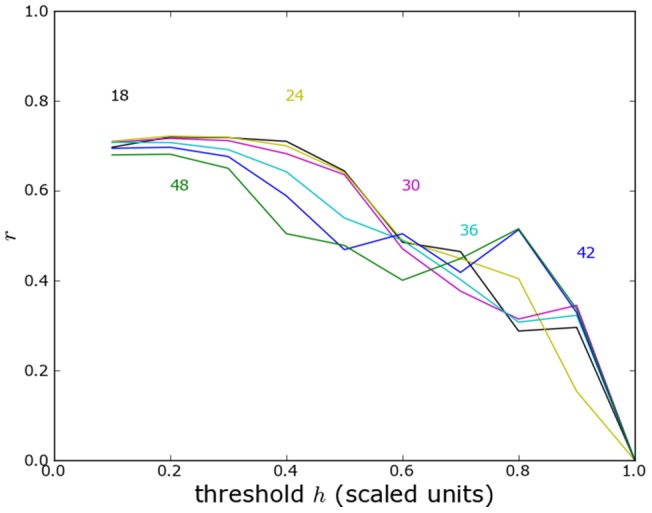
Yugoslavia correlation analysis including Slovenia and Macedonia without boundaries. As in Fig. 14 but including Slovenia and Macedonia.

**Figure 18:**
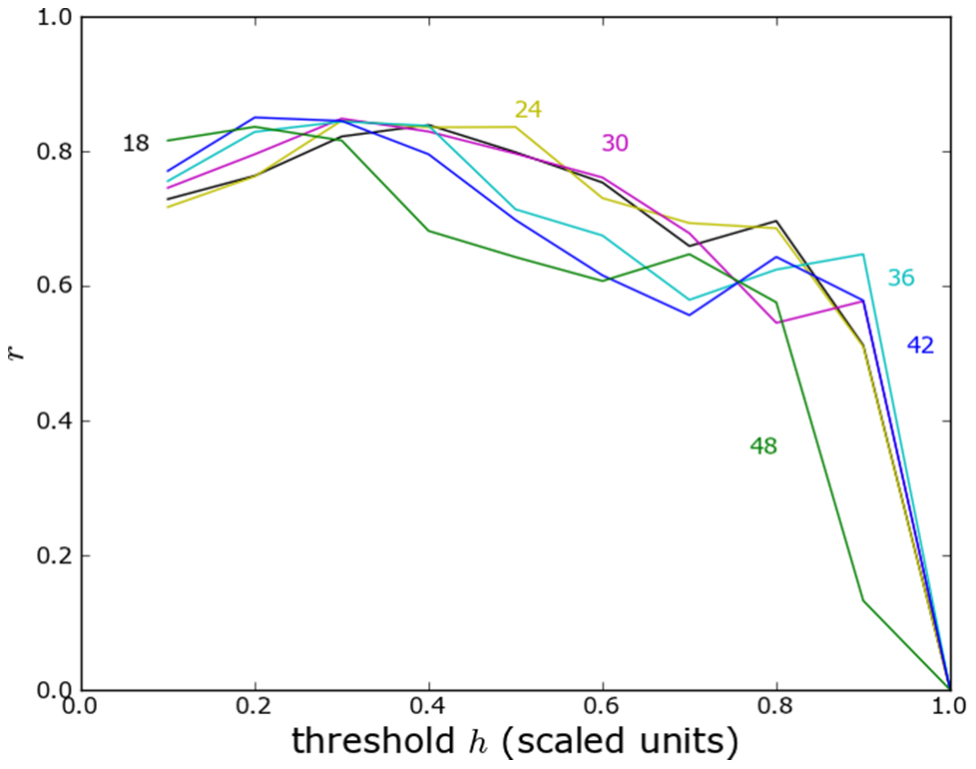
Yugoslavia correlation analysis including Slovenia and Macedonia with political boundaries. As in Fig. 17 but including the effect of political boundaries.

We also provide a similar analysis of the former Yugoslavia including Macedonia and Slovenia, without (Fig. 17) and with (Fig. 18) political boundaries. Without political boundaries the agreement of predicted and reported violence is dramatically reduced.
